# [4-(Bromo­methyl)­benz­yl]triphenyl­phospho­nium bromide acetonitrile monosolvate

**DOI:** 10.1107/S1600536812042341

**Published:** 2012-10-24

**Authors:** Benjamin P. Burke, Peter Greenman, Adam M. Smith, Stephen J. Archibald

**Affiliations:** a Department of Chemistry, University of Hull, Hull, HU6 7RX, UK

## Abstract

In the title compound, C_26_H_23_BrP^+^·Br^−^·C_2_H_3_N, the dihedral angles between the plane of the benzylic phenyl ring attached to the P atom and the planes of the three directly attached phenyl rings are 34.04 (12), 45.48 (13) and 87.18 (9)°. In the crystal, centrosymmetric pairs of cations and anions are linked into dimeric aggregates *via* C—H⋯Br hydrogen bonds. There is also a C—H⋯N hydrogen bond to the acetonitrile solvent mol­ecule.

## Related literature

For background to the biological activity of alkyl­triphenyl­phospho­nium derivatives, see: Modica-Napolitano & Aprille (2001 ▶); Modica-Napolitano & Singh (2002 ▶); Wang *et al.* (2007 ▶); Kim *et al.* (2008 ▶, 2012 ▶); Madar *et al.* (2007 ▶). For the synthesis of triphenyl­phospho­nium salts, see: Wang *et al.* (2007 ▶). 
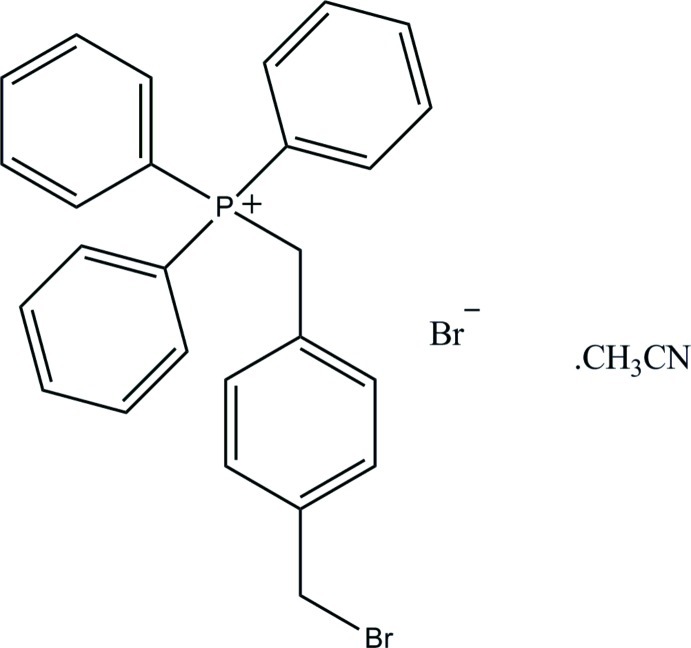


## Experimental

### 

#### Crystal data

C_26_H_23_BrP·Br·C_2_H_3_N
*M* *_r_* = 567.29
Triclinic, 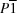

*a* = 9.588 (2) Å
*b* = 12.333 (3) Å
*c* = 12.393 (3) Å
α = 74.961 (19)°
β = 70.051 (18)°
γ = 69.293 (19)°
*V* = 1272.4 (5) Å^3^
*Z* = 2
Mo *K*α radiation
μ = 3.26 mm^−1^
*T* = 150 K
0.2 × 0.2 × 0.1 mm

#### Data collection

Stoe IPDSII diffractometer
Absorption correction: integration (*X-AREA*; Stoe & Cie, 2002 ▶) *T*_min_ = 0.336, *T*_max_ = 0.661
9761 measured reflections
4473 independent reflections
2659 reflections with *I* > 2σ(*I*)
*R*_int_ = 0.069

#### Refinement

*R*[*F* ^2^ > 2σ(*F* ^2^)] = 0.029
*wR*(*F* ^2^) = 0.051
*S* = 0.68
4473 reflections
290 parameters
H-atom parameters constrained
Δρ_max_ = 0.41 e Å^−3^
Δρ_min_ = −0.36 e Å^−3^

### 

Data collection: *X-AREA* (Stoe & Cie, 2002 ▶); cell refinement: *X-AREA*; data reduction: *X-RED* (Stoe & Cie, 2002 ▶); program(s) used to solve structure: *SHELXS97* (Sheldrick, 2008 ▶); program(s) used to refine structure: *SHELXL97* (Sheldrick, 2008 ▶); molecular graphics: *ORTEP-3* (Farrugia, 1997 ▶); software used to prepare material for publication: *SHELXL97* and *WinGX* (Farrugia, 1999 ▶).

## Supplementary Material

Crystal structure: contains datablock(s) I, New_Global_Publ_Block. DOI: 10.1107/S1600536812042341/rz5012sup1.cif

Structure factors: contains datablock(s) I. DOI: 10.1107/S1600536812042341/rz5012Isup2.hkl

Supplementary material file. DOI: 10.1107/S1600536812042341/rz5012Isup3.cdx

Supplementary material file. DOI: 10.1107/S1600536812042341/rz5012Isup4.cml

Additional supplementary materials:  crystallographic information; 3D view; checkCIF report

## Figures and Tables

**Table 1:** Hydrogen-bond geometry (Å, °)

*D*—H⋯*A*	*D*—H	H⋯*A*	*D*⋯*A*	*D*—H⋯*A*
C1—H1*B*⋯N1^i^	0.99	2.60	3.488 (6)	150
C8—H8*A*⋯Br2^ii^	0.99	2.64	3.625 (4)	172
C8—H8*B*⋯Br2^iii^	0.99	2.79	3.753 (3)	166
C20—H20⋯Br2^iii^	0.95	2.81	3.746 (4)	169
C28—H28*C*⋯Br2	0.98	2.69	3.673 (5)	176

Symmetry codes: (i) 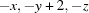; (ii) 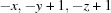; (iii) 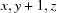.

**Fig. 1.**
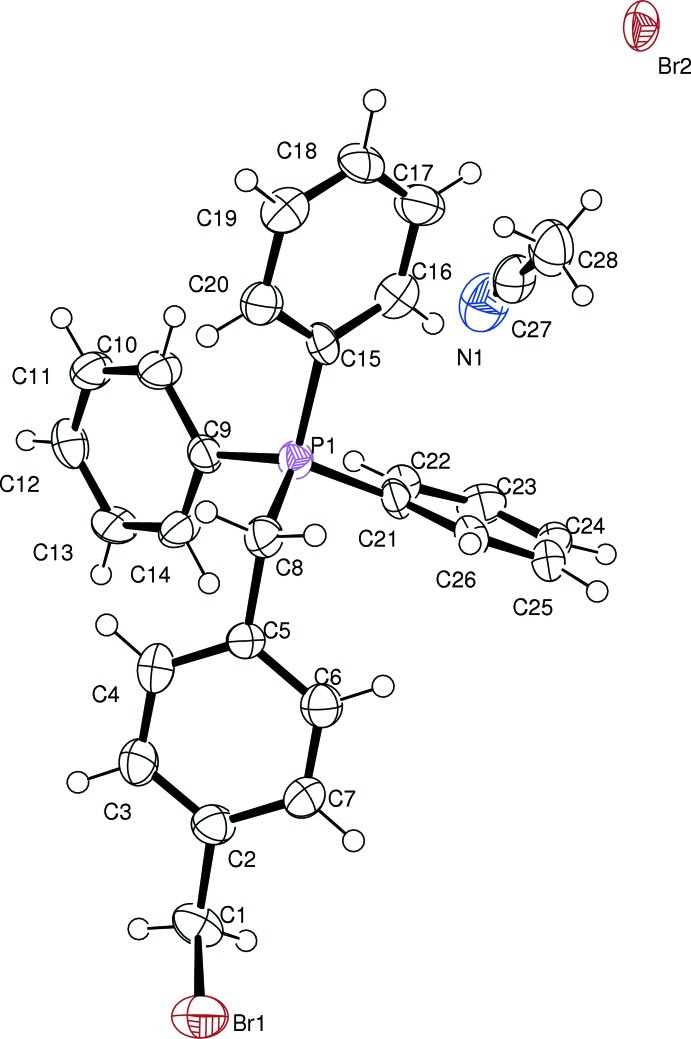
An ORTEP plot of the title compound using 50% probability ellipsoids with all non-H atoms labelled.

**Fig. 2.**
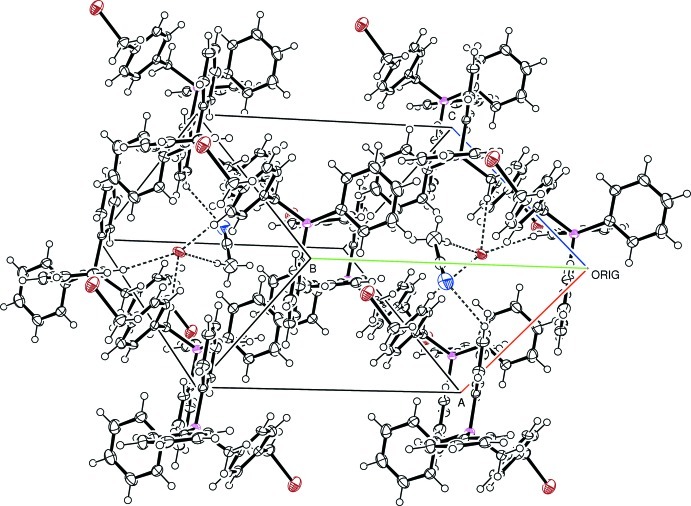
The packing in the unit cell with displacement ellipsoids drawn at the 50% probability level. Intermolecular hydrogen bonds are shown as dashed lines.

## supplementary crystallographic information

### Comment

Triphenylphosphonium cations are known to accumulate in cancer cell mitochondria due to a significant increase in mitochondrial transmembrane potential between normal epithelial cells and carcinoma cells, causing a potential tenfold higher accumulation of cationic compounds in carcinoma cells (Modica-Napolitano *et al.*, 2001; Modica-Napolitano *et al.*, 2002). Increase in uptake in myocardial cells also gives the potential for use as heart targeting agents (Kim *et al.*, 2012). (4-Bromomethylbenzyl)triphenylphosphonium bromide has been used as a precursor to synthesize a ^64^Cu radiolabelled molecule for potential cancer detection by positron emission tomography (Wang *et al.*, 2007). Related compounds have also been radiolabelled with the ^18^F isotope (Kim *et al.*, 2012; Madar *et al.*, 2007).

In the cation of the title compound (Fig. 1), the dihedral angles formed by the C2–C7 benzene ring with the C9–C14, C15–C20 and C21–C26 phenyl rings are 34.04 (12), 45.48 (13) and 87.18 (9)°, respectively. In the crystal (Fig. 2), centrosymmetric pairs of ions are linked through C—H···Br hydrogen bonds (Table 1). The dimeric aggregates interact with the acetonitrile solvent molecules by C—H···N hydrogen bonds.

### Experimental

The synthetic procedure was completed following literature methods (Wang *et al.*, 2007). Triphenylphosphine (2 g, 7.73 mmol) in toluene (25 ml) was added dropwise to a stirred solution of α,α-dibromo-*p*-xylene (2.01 g, 7.73 mmol) in toluene (25 ml). The mixture was heated under reflux for 18 h. The reaction was cooled to room temperature, filtered and washed with toluene (20 ml) and diethyl ether (20 ml) to as a white solid (3.35 g, 82%). ^1^H-NMR (CDCl_3_): δ 4.39 (s, 2H, CH_2_—Br), 5.48 (d, 2H, CH_2_—P, J = 14.7 Hz), 7.10 (s, 4H, CH—Ar), 7.61 (m, 6H, CH—Ar), 7.76 (m, 9H, CH—Ar). ^13^C-NMR (CDCl_3_): δ 30.22 (CH_2_—P), 30.68 (CH_2_—P), 32.98 (CH_2_—P), 117.35 (CH—Ar), 118.21 (CH—Ar), 127.75 (C—Ar), 127.83 (C—Ar), 129.48 (CH—Ar), 130.18 (CH—Ar), 130.30 (CH—Ar), 132.10 (CH—Ar), 134.56 (CH—Ar), 135.09 (CH—Ar), 138.17 (C—Ar). ^31^P-NMR (CDCl_3_): δ 24.09 (s, PPh_3_). The crystals were grown by vapour diffusion of diethyl ether into an acetonitrile solution of the title compound at room temperature.

### Refinement

All H atoms were positioned geometrically and treated as riding, with C—H = 0.95 Å and *U*_iso_(H) = 1.2*U*_eq_(C) for aromatic H atoms, with C—H = 0.99 Å and *U*_iso_(H) = 1.2*U*_eq_(C) for methylene H atoms and with C—H = 0.98 Å and *U*_iso_(H) = 1.5*U*_eq_(C) for methyl H atoms.

### Crystal data

**Table:**

C_26_H_23_BrP·Br·C_2_H_3_N	*Z* = 2
*M**_r_* = 567.29	*F*(000) = 572
Triclinic, *P*1	*D*_x_ = 1.481 Mg m^−^^3^
Hall symbol: -P 1	Mo *K*α radiation, λ = 0.71073 Å
*a* = 9.588 (2) Å	Cell parameters from 8439 reflections
*b* = 12.333 (3) Å	θ = 2.5–31.3°
*c* = 12.393 (3) Å	µ = 3.26 mm^−^^1^
α = 74.961 (19)°	*T* = 150 K
β = 70.051 (18)°	Block, colourless
γ = 69.293 (19)°	0.2 × 0.2 × 0.1 mm
*V* = 1272.4 (5) Å^3^	

### Data collection

**Table:**

Stoe IPDSII diffractometer	4473 independent reflections
Radiation source: sealed X-ray tube, 12 x 0.4 mm long-fine focus	2659 reflections with *I* > 2σ(*I*)
Plane graphite monochromator	*R*_int_ = 0.069
Detector resolution: 6.67 pixels mm^-1^	θ_max_ = 25.0°, θ_min_ = 2.5°
rotation method scans	*h* = −11→11
Absorption correction: integration (*X-AREA*; Stoe & Cie, 2002)	*k* = −14→14
*T*_min_ = 0.336, *T*_max_ = 0.661	*l* = −14→14
9761 measured reflections	

### Refinement

**Table:**

Refinement on *F*^2^	Primary atom site location: structure-invariant direct methods
Least-squares matrix: full	Secondary atom site location: difference Fourier map
*R*[*F*^2^ > 2σ(*F*^2^)] = 0.029	Hydrogen site location: inferred from neighbouring sites
*wR*(*F*^2^) = 0.051	H-atom parameters constrained
*S* = 0.68	*w* = 1/[σ^2^(*F*_o_^2^) + (0.006*P*)^2^] where *P* = (*F*_o_^2^ + 2*F*_c_^2^)/3
4473 reflections	(Δ/σ)_max_ = 0.001
290 parameters	Δρ_max_ = 0.41 e Å^−^^3^
0 restraints	Δρ_min_ = −0.36 e Å^−^^3^

### Special details

**Table:**

Geometry. All e.s.d.'s (except the e.s.d. in the dihedral angle between two l.s. planes) are estimated using the full covariance matrix. The cell e.s.d.'s are taken into account individually in the estimation of e.s.d.'s in distances, angles and torsion angles; correlations between e.s.d.'s in cell parameters are only used when they are defined by crystal symmetry. An approximate (isotropic) treatment of cell e.s.d.'s is used for estimating e.s.d.'s involving l.s. planes.
Refinement. Refinement of *F*^2^ against ALL reflections. The weighted *R*-factor *wR* and goodness of fit *S* are based on *F*^2^, conventional *R*-factors *R* are based on *F*, with *F* set to zero for negative *F*^2^. The threshold expression of *F*^2^ > σ(*F*^2^) is used only for calculating *R*-factors(gt) *etc*. and is not relevant to the choice of reflections for refinement. *R*-factors based on *F*^2^ are statistically about twice as large as those based on *F*, and *R*- factors based on ALL data will be even larger.

### Fractional atomic coordinates and isotropic or equivalent isotropic displacement parameters (Å^2^)

**Table:**

	*x*	*y*	*z*	*U*_iso_*/*U*_eq_	
Br1	−0.52982 (5)	1.49983 (4)	0.20248 (4)	0.04070 (13)	
P1	0.06715 (11)	0.85342 (8)	0.29654 (7)	0.0195 (2)	
C1	−0.3828 (5)	1.3828 (3)	0.1005 (3)	0.0329 (9)	
H1A	−0.4412	1.3555	0.0661	0.04*	
H1B	−0.3108	1.4201	0.0363	0.04*	
C2	−0.2931 (4)	1.2814 (3)	0.1680 (3)	0.0236 (8)	
C3	−0.1400 (5)	1.2690 (3)	0.1579 (3)	0.0294 (9)	
H3	−0.0933	1.3272	0.1085	0.035*	
C4	−0.0535 (4)	1.1727 (3)	0.2190 (3)	0.0241 (8)	
H4	0.0523	1.1643	0.2091	0.029*	
C5	−0.1216 (4)	1.0885 (3)	0.2945 (3)	0.0218 (8)	
C6	−0.2768 (4)	1.1031 (3)	0.3071 (3)	0.0217 (8)	
H6	−0.3252	1.0469	0.3593	0.026*	
C7	−0.3611 (4)	1.1972 (3)	0.2453 (3)	0.0251 (8)	
H7	−0.4668	1.2053	0.2552	0.03*	
C8	−0.0296 (4)	0.9850 (3)	0.3620 (3)	0.0227 (8)	
H8A	−0.1004	0.9652	0.4387	0.027*	
H8B	0.0498	1.0093	0.3762	0.027*	
C9	0.2092 (4)	0.8782 (3)	0.1619 (3)	0.0204 (8)	
C10	0.3660 (4)	0.8213 (3)	0.1497 (3)	0.0244 (8)	
H10	0.3989	0.7697	0.2138	0.029*	
C11	0.4742 (5)	0.8391 (3)	0.0450 (3)	0.0318 (9)	
H11	0.5812	0.7988	0.0362	0.038*	
C12	0.4249 (5)	0.9165 (3)	−0.0472 (3)	0.0302 (9)	
H12	0.4989	0.9296	−0.1194	0.036*	
C13	0.2709 (5)	0.9743 (3)	−0.0353 (3)	0.0309 (9)	
H13	0.2387	1.0275	−0.099	0.037*	
C14	0.1624 (4)	0.9557 (3)	0.0685 (3)	0.0244 (8)	
H14	0.0555	0.9957	0.0763	0.029*	
C15	0.1567 (4)	0.7414 (3)	0.3975 (3)	0.0198 (8)	
C16	0.1466 (4)	0.6276 (3)	0.4163 (3)	0.0264 (9)	
H16	0.0899	0.6102	0.3767	0.032*	
C17	0.2181 (5)	0.5399 (3)	0.4921 (3)	0.0320 (9)	
H17	0.2102	0.4627	0.5047	0.038*	
C18	0.3018 (4)	0.5651 (3)	0.5497 (3)	0.0290 (9)	
H18	0.3519	0.5049	0.6014	0.035*	
C19	0.3122 (5)	0.6764 (3)	0.5322 (3)	0.0269 (9)	
H19	0.3684	0.6931	0.5728	0.032*	
C20	0.2417 (4)	0.7656 (3)	0.4559 (3)	0.0251 (8)	
H20	0.251	0.8423	0.4435	0.03*	
C21	−0.0684 (4)	0.8008 (3)	0.2692 (3)	0.0225 (8)	
C22	−0.0205 (4)	0.7420 (3)	0.1738 (3)	0.0287 (9)	
H22	0.0792	0.736	0.1202	0.034*	
C23	−0.1217 (5)	0.6925 (3)	0.1590 (3)	0.0352 (10)	
H23	−0.0903	0.6515	0.0955	0.042*	
C24	−0.2656 (5)	0.7026 (3)	0.2355 (3)	0.0351 (10)	
H24	−0.3341	0.6695	0.2239	0.042*	
C25	−0.3130 (5)	0.7606 (3)	0.3295 (3)	0.0348 (10)	
H25	−0.4137	0.7676	0.3817	0.042*	
C26	−0.2129 (4)	0.8083 (3)	0.3474 (3)	0.0269 (9)	
H26	−0.2438	0.846	0.4131	0.032*	
N1	0.1911 (5)	0.4067 (4)	0.0535 (4)	0.0620 (12)	
C27	0.1653 (5)	0.3903 (4)	0.1509 (4)	0.0397 (11)	
C28	0.1253 (6)	0.3696 (4)	0.2764 (4)	0.0597 (14)	
H28A	0.0123	0.3888	0.3079	0.089*	
H28B	0.1638	0.419	0.3028	0.089*	
H28C	0.1726	0.2868	0.3037	0.089*	
Br2	0.31729 (5)	0.05853 (4)	0.36399 (3)	0.03526 (12)	

### Atomic displacement parameters (Å^2^)

**Table:**

	*U*^11^	*U*^22^	*U*^33^	*U*^12^	*U*^13^	*U*^23^
Br1	0.0414 (3)	0.0292 (3)	0.0486 (3)	0.0017 (2)	−0.0175 (2)	−0.0113 (2)
P1	0.0172 (5)	0.0218 (5)	0.0185 (5)	−0.0050 (4)	−0.0032 (4)	−0.0050 (4)
C1	0.039 (3)	0.031 (2)	0.024 (2)	−0.006 (2)	−0.0057 (18)	−0.0067 (17)
C2	0.025 (2)	0.022 (2)	0.0220 (18)	−0.0026 (18)	−0.0049 (16)	−0.0094 (16)
C3	0.038 (3)	0.027 (2)	0.025 (2)	−0.017 (2)	−0.0027 (18)	−0.0034 (17)
C4	0.017 (2)	0.030 (2)	0.0236 (19)	−0.0083 (18)	0.0004 (15)	−0.0063 (17)
C5	0.023 (2)	0.018 (2)	0.0249 (19)	−0.0045 (17)	−0.0050 (16)	−0.0086 (15)
C6	0.024 (2)	0.019 (2)	0.0227 (18)	−0.0099 (17)	−0.0035 (16)	−0.0041 (15)
C7	0.022 (2)	0.021 (2)	0.032 (2)	−0.0027 (18)	−0.0074 (17)	−0.0092 (17)
C8	0.025 (2)	0.021 (2)	0.0215 (18)	−0.0082 (18)	−0.0031 (16)	−0.0049 (16)
C9	0.018 (2)	0.023 (2)	0.0218 (18)	−0.0050 (17)	−0.0056 (15)	−0.0085 (15)
C10	0.021 (2)	0.027 (2)	0.0236 (19)	−0.0053 (17)	−0.0071 (16)	−0.0015 (16)
C11	0.022 (2)	0.038 (3)	0.033 (2)	−0.0071 (19)	−0.0012 (17)	−0.0119 (19)
C12	0.035 (3)	0.036 (2)	0.0193 (19)	−0.017 (2)	0.0005 (17)	−0.0064 (17)
C13	0.042 (3)	0.030 (2)	0.0192 (19)	−0.010 (2)	−0.0091 (18)	−0.0004 (17)
C14	0.020 (2)	0.030 (2)	0.0204 (18)	−0.0045 (18)	−0.0034 (16)	−0.0068 (16)
C15	0.017 (2)	0.021 (2)	0.0167 (17)	−0.0030 (16)	0.0007 (14)	−0.0062 (14)
C16	0.027 (2)	0.030 (2)	0.0215 (19)	−0.0077 (19)	−0.0064 (17)	−0.0056 (16)
C17	0.037 (3)	0.021 (2)	0.035 (2)	−0.007 (2)	−0.0100 (19)	−0.0015 (18)
C18	0.022 (2)	0.028 (2)	0.0245 (19)	0.0061 (18)	−0.0072 (17)	−0.0014 (17)
C19	0.022 (2)	0.032 (2)	0.024 (2)	−0.0021 (18)	−0.0066 (17)	−0.0071 (17)
C20	0.026 (2)	0.023 (2)	0.0250 (19)	−0.0064 (18)	−0.0065 (16)	−0.0043 (16)
C21	0.023 (2)	0.023 (2)	0.0210 (18)	−0.0058 (17)	−0.0085 (16)	−0.0008 (15)
C22	0.029 (2)	0.034 (2)	0.0248 (19)	−0.0103 (19)	−0.0040 (17)	−0.0099 (17)
C23	0.046 (3)	0.037 (3)	0.032 (2)	−0.017 (2)	−0.016 (2)	−0.0062 (19)
C24	0.034 (3)	0.031 (3)	0.046 (3)	−0.013 (2)	−0.021 (2)	0.002 (2)
C25	0.025 (2)	0.033 (2)	0.046 (2)	−0.013 (2)	−0.0068 (19)	−0.004 (2)
C26	0.024 (2)	0.022 (2)	0.031 (2)	−0.0029 (18)	−0.0049 (17)	−0.0075 (17)
N1	0.060 (3)	0.072 (3)	0.065 (3)	−0.033 (3)	−0.010 (2)	−0.020 (2)
C27	0.033 (3)	0.039 (3)	0.051 (3)	−0.016 (2)	−0.002 (2)	−0.020 (2)
C28	0.065 (4)	0.063 (4)	0.053 (3)	−0.026 (3)	−0.003 (3)	−0.021 (3)
Br2	0.0345 (3)	0.0474 (3)	0.0261 (2)	−0.0247 (2)	0.00201 (18)	−0.0056 (2)

### Geometric parameters (Å, º)

**Table:**

Br1—C1	1.990 (4)	C13—H13	0.95
P1—C15	1.791 (3)	C14—H14	0.95
P1—C9	1.792 (3)	C15—C16	1.394 (4)
P1—C21	1.800 (3)	C15—C20	1.401 (4)
P1—C8	1.805 (3)	C16—C17	1.382 (5)
C1—C2	1.480 (5)	C16—H16	0.95
C1—H1A	0.99	C17—C18	1.391 (5)
C1—H1B	0.99	C17—H17	0.95
C2—C3	1.386 (5)	C18—C19	1.367 (5)
C2—C7	1.400 (5)	C18—H18	0.95
C3—C4	1.392 (5)	C19—C20	1.390 (5)
C3—H3	0.95	C19—H19	0.95
C4—C5	1.392 (5)	C20—H20	0.95
C4—H4	0.95	C21—C26	1.381 (5)
C5—C6	1.393 (5)	C21—C22	1.405 (4)
C5—C8	1.504 (5)	C22—C23	1.394 (5)
C6—C7	1.373 (5)	C22—H22	0.95
C6—H6	0.95	C23—C24	1.366 (5)
C7—H7	0.95	C23—H23	0.95
C8—H8A	0.99	C24—C25	1.384 (5)
C8—H8B	0.99	C24—H24	0.95
C9—C10	1.389 (5)	C25—C26	1.388 (5)
C9—C14	1.390 (4)	C25—H25	0.95
C10—C11	1.379 (5)	C26—H26	0.95
C10—H10	0.95	N1—C27	1.123 (5)
C11—C12	1.387 (5)	C27—C28	1.446 (6)
C11—H11	0.95	C28—H28A	0.98
C12—C13	1.369 (5)	C28—H28B	0.98
C12—H12	0.95	C28—H28C	0.98
C13—C14	1.375 (5)		
			
C15—P1—C9	110.46 (16)	C12—C13—H13	119.9
C15—P1—C21	107.13 (16)	C14—C13—H13	119.9
C9—P1—C21	108.45 (15)	C13—C14—C9	120.0 (4)
C15—P1—C8	108.01 (14)	C13—C14—H14	120
C9—P1—C8	111.25 (16)	C9—C14—H14	120
C21—P1—C8	111.47 (17)	C16—C15—C20	119.0 (3)
C2—C1—Br1	110.4 (2)	C16—C15—P1	120.2 (2)
C2—C1—H1A	109.6	C20—C15—P1	120.7 (3)
Br1—C1—H1A	109.6	C17—C16—C15	120.7 (3)
C2—C1—H1B	109.6	C17—C16—H16	119.6
Br1—C1—H1B	109.6	C15—C16—H16	119.6
H1A—C1—H1B	108.1	C16—C17—C18	119.7 (3)
C3—C2—C7	118.2 (3)	C16—C17—H17	120.2
C3—C2—C1	120.6 (3)	C18—C17—H17	120.2
C7—C2—C1	121.2 (3)	C19—C18—C17	120.1 (3)
C2—C3—C4	121.1 (3)	C19—C18—H18	119.9
C2—C3—H3	119.5	C17—C18—H18	119.9
C4—C3—H3	119.5	C18—C19—C20	121.0 (3)
C3—C4—C5	120.2 (3)	C18—C19—H19	119.5
C3—C4—H4	119.9	C20—C19—H19	119.5
C5—C4—H4	119.9	C19—C20—C15	119.5 (3)
C4—C5—C6	118.6 (3)	C19—C20—H20	120.3
C4—C5—C8	120.5 (3)	C15—C20—H20	120.3
C6—C5—C8	120.9 (3)	C26—C21—C22	120.3 (3)
C7—C6—C5	121.0 (3)	C26—C21—P1	120.1 (3)
C7—C6—H6	119.5	C22—C21—P1	119.3 (3)
C5—C6—H6	119.5	C23—C22—C21	118.9 (3)
C6—C7—C2	120.8 (3)	C23—C22—H22	120.5
C6—C7—H7	119.6	C21—C22—H22	120.5
C2—C7—H7	119.6	C24—C23—C22	120.3 (3)
C5—C8—P1	116.8 (2)	C24—C23—H23	119.9
C5—C8—H8A	108.1	C22—C23—H23	119.9
P1—C8—H8A	108.1	C23—C24—C25	120.9 (3)
C5—C8—H8B	108.1	C23—C24—H24	119.6
P1—C8—H8B	108.1	C25—C24—H24	119.6
H8A—C8—H8B	107.3	C24—C25—C26	119.9 (4)
C10—C9—C14	119.4 (3)	C24—C25—H25	120.1
C10—C9—P1	120.8 (3)	C26—C25—H25	120.1
C14—C9—P1	119.8 (3)	C21—C26—C25	119.7 (3)
C11—C10—C9	120.4 (3)	C21—C26—H26	120.1
C11—C10—H10	119.8	C25—C26—H26	120.1
C9—C10—H10	119.8	N1—C27—C28	177.5 (5)
C10—C11—C12	119.2 (4)	C27—C28—H28A	109.5
C10—C11—H11	120.4	C27—C28—H28B	109.5
C12—C11—H11	120.4	H28A—C28—H28B	109.5
C13—C12—C11	120.7 (4)	C27—C28—H28C	109.5
C13—C12—H12	119.6	H28A—C28—H28C	109.5
C11—C12—H12	119.6	H28B—C28—H28C	109.5
C12—C13—C14	120.3 (3)		
			
Br1—C1—C2—C3	−104.0 (3)	P1—C9—C14—C13	−179.5 (2)
Br1—C1—C2—C7	74.5 (3)	C9—P1—C15—C16	−101.6 (3)
C7—C2—C3—C4	2.8 (5)	C21—P1—C15—C16	16.3 (3)
C1—C2—C3—C4	−178.6 (3)	C8—P1—C15—C16	136.5 (3)
C2—C3—C4—C5	−2.0 (5)	C9—P1—C15—C20	76.4 (3)
C3—C4—C5—C6	0.1 (5)	C21—P1—C15—C20	−165.6 (3)
C3—C4—C5—C8	−179.2 (3)	C8—P1—C15—C20	−45.4 (3)
C4—C5—C6—C7	1.0 (5)	C20—C15—C16—C17	0.5 (5)
C8—C5—C6—C7	−179.7 (3)	P1—C15—C16—C17	178.6 (3)
C5—C6—C7—C2	−0.1 (5)	C15—C16—C17—C18	−0.3 (6)
C3—C2—C7—C6	−1.8 (5)	C16—C17—C18—C19	0.5 (6)
C1—C2—C7—C6	179.7 (3)	C17—C18—C19—C20	−0.9 (6)
C4—C5—C8—P1	−92.4 (3)	C18—C19—C20—C15	1.0 (6)
C6—C5—C8—P1	88.4 (4)	C16—C15—C20—C19	−0.8 (5)
C15—P1—C8—C5	−176.2 (3)	P1—C15—C20—C19	−178.9 (3)
C9—P1—C8—C5	62.4 (3)	C15—P1—C21—C26	80.5 (3)
C21—P1—C8—C5	−58.8 (3)	C9—P1—C21—C26	−160.3 (3)
C15—P1—C9—C10	−5.0 (3)	C8—P1—C21—C26	−37.5 (3)
C21—P1—C9—C10	−122.1 (3)	C15—P1—C21—C22	−93.5 (3)
C8—P1—C9—C10	114.9 (3)	C9—P1—C21—C22	25.8 (3)
C15—P1—C9—C14	175.2 (3)	C8—P1—C21—C22	148.6 (3)
C21—P1—C9—C14	58.1 (3)	C26—C21—C22—C23	0.6 (5)
C8—P1—C9—C14	−64.9 (3)	P1—C21—C22—C23	174.5 (3)
C14—C9—C10—C11	−1.5 (5)	C21—C22—C23—C24	0.9 (6)
P1—C9—C10—C11	178.7 (2)	C22—C23—C24—C25	−0.9 (6)
C9—C10—C11—C12	1.4 (5)	C23—C24—C25—C26	−0.5 (6)
C10—C11—C12—C13	−0.4 (5)	C22—C21—C26—C25	−1.9 (6)
C11—C12—C13—C14	−0.4 (5)	P1—C21—C26—C25	−175.8 (3)
C12—C13—C14—C9	0.3 (5)	C24—C25—C26—C21	1.9 (6)
C10—C9—C14—C13	0.7 (5)		

### Hydrogen-bond geometry (Å, º)

**Table:**

*D*—H···*A*	*D*—H	H···*A*	*D*···*A*	*D*—H···*A*
C1—H1*B*···N1^i^	0.99	2.60	3.488 (6)	150
C8—H8*A*···Br2^ii^	0.99	2.64	3.625 (4)	172
C8—H8*B*···Br2^iii^	0.99	2.79	3.753 (3)	166
C20—H20···Br2^iii^	0.95	2.81	3.746 (4)	169
C28—H28*C*···Br2	0.98	2.69	3.673 (5)	176

Symmetry codes: (i) −*x*, −*y*+2, −*z*; (ii) −*x*, −*y*+1, −*z*+1; (iii) *x*, *y*+1, *z*.
